# Real-world use of sunitinib in Japanese patients with pancreatic neuroendocrine tumors: results from a post-marketing surveillance study

**DOI:** 10.1007/s00280-018-3724-3

**Published:** 2018-11-09

**Authors:** Kazuo Sato, Yasuharu Toyoshima, Shiho Moriyama, Yutaka Endo, Tetsuhide Ito, Emiko Ohki

**Affiliations:** 1 Pfizer Japan, Tokyo, Japan; 2 Fukuoka Sanno Hospital, International University of Health and Welfare, Tokyo, Japan

**Keywords:** Pancreatic neuroendocrine tumors; Japan, Sunitinib, Safety, Objective response rate

## Abstract

**Background:**

Sunitinib is approved for the treatment of progressive, well-differentiated pancreatic neuroendocrine tumors (pNETs) in patients with unresectable, locally advanced or metastatic disease. Safety and efficacy data in Japanese patients are limited. We report outcomes from a post-marketing surveillance study of sunitinib treatment in Japanese patients.

**Methods:**

Sunitinib 37.5 mg once daily was orally administered in Japanese patients aged ≥ 15 years with pNETs. The primary endpoints included adverse events (AEs) occurring during the observation period of 168 days and objective response rate (ORR).

**Results:**

Sunitinib was administered in 62 patients with pNETs. The median duration of treatment was 165 days. At 168 days from the start of treatment, 31 patients were still receiving sunitinib treatment and treatment continuation rate was 50.0%. Of the 31 patients who discontinued treatment, 18 (58.1%) discontinued because of AEs and 16 (51.6%) patients discontinued due to insufficient clinical effect. Of the 18 patients who discontinued due to AEs, 10 did so within 42 days of treatment initiation. The most common all-grade AEs were platelet count decreased (33.9%), diarrhea (29.0%), neutrophil count decreased (27.4%), hypertension (24.2%), and palmar-plantar erythrodysesthesia syndrome (24.2%). In the 51 patients eligible for the efficacy analysis, ORR was 13.7% (95% confidence interval, 5.7–26.3) and clinical benefit rate was 70.6%.

**Conclusions:**

There were no new safety concerns in real-world use of sunitinib in Japanese patients with pNETs. The short treatment duration likely led to low tumor response. Appropriate AEs management through dose interruption/reduction is essential for sunitinib treatment success in this patient population.

## Introduction

Pancreatic neuroendocrine tumors (pNETs) are considered rare, but their incidence worldwide is increasing annually [1–4]. In Japan alone, a 20% increase in the number of patients treated for pNETs was recorded in the year 2010 compared with 2005 [5]. The overall prevalence of pNETs in Japan in 2010 was 2.69 per 100,000 population [5].

Surgery is the standard of care for localized pNETs [6]; however, many patients are diagnosed at late stage with advanced or unresectable metastatic disease whereby surgery is not always an option [4, 7]. Prognosis for patients with pNETs is dependent on the histology and disease stage at diagnosis. For patients with well- or moderately-differentiated pNETs, 5-year survival rate is 79% for localized disease, 62% for regional disease, and 27% in patients with distant metastases [3]. The outcome in patients with poorly differentiated pNETs is worse, depending on the disease stage [3].

The clinical outcomes in patients with unresectable metastatic pNETs have improved with the introduction of new therapies, including everolimus (AFINITOR; Novartis Pharmaceuticals, East Hanover, NJ, USA), sunitinib (SUTENT; Pfizer Inc, New York, NY, USA), and streptozotocin (ZANOSAR; Teva Pharmaceuticals, North Wales, UK) [8–12]. All three agents are available for use in Japan.

Sunitinib is a potent inhibitor of multiple tyrosine kinase receptors, including the vascular endothelial growth factor receptors (VEGFR) and platelet-derived growth factor receptors (PDGFR) that are essential for pNETs proliferation and angiogenesis [13–15]. In a randomized phase III trial, sunitinib (37.5 mg once daily) improved progression-free survival compared with placebo in patients with advanced, well-differentiated pNETs [hazard ratio (HR) 0.42; 95% confidence interval (CI) 0.26–0.66; *P* = 0.0001] [8]. Commonly reported adverse events (AEs) were manageable and consistent with the known safety profile of sunitinib. An updated analysis of overall survival (OS) at 5 years after study closure favored sunitinib vs. placebo (HR 0.73; 95% CI 0.50–1.06; *P* = 0.094), despite that most patients (69%) in the placebo arm crossed over to sunitinib [16].

Sunitinib has also demonstrated antitumor activity and a manageable safety profile in Japanese patients with pNETs [11]. In a phase II trial in 12 Japanese patients, sunitinib demonstrated antitumor activity, with an objective response rate (ORR) of 50% (95% CI 21.1–78.9), a median PFS 16.8 months (95% CI 9.3–26.2), and a safety profile similar to that shown in the global trial [11, 17]. As a result, sunitinib was approved globally, including Japan, for the treatment of progressive, well-differentiated pNETs in patients with unresectable, locally advanced or metastatic disease [18].

At the time of sunitinib approval for treatment of pNETs in Japan, safety and efficacy data in Japanese patients were limited. Therefore, the Japan Pharmaceuticals and Medical Devices Agency requested the conduct of a post-marketing surveillance (PMS) study to expand the safety database and ensure appropriate use of sunitinib in the Japanese population. Here, we report the outcomes from this PMS study, including adverse drug reactions and efficacy associated with sunitinib in Japanese patients with pNETs, and how sunitinib treatment is managed in clinical practice in Japan.

## Methods

### Study design and treatment

This was a PMS study of sunitinib in patients aged ≥ 15 years with pNETs. Sunitinib was orally administered at a starting dose of 37.5 mg once daily (using 12.5 mg capsules). Dose increase (to a maximum of 50 mg once daily) or decrease were permitted depending on patient tolerance. The study was conducted between August 10, 2012 (the date of sunitinib approval in Japan) and February 2017 in 20 centers in Japan specializing in the treatment of pNETs.

The investigation consisted of two periods, registration and observation. The registration period continued until the target number of 60 patients was achieved or for 4 years, whichever was earlier. The observation period was 168 days (24 weeks) from the first day of sunitinib administration. Patients were observed until treatment completion or discontinuation. During the observation period, investigators were required to document information about sunitinib administration (daily dose and frequency, administration period, reasons for dose modifications) and discontinuation, with reasons for discontinuation.

Safety assessments included AEs (graded according to the National Cancer Institute Common Terminology Criteria for Adverse Events v4.0), laboratory assessments (hematology and biochemistry), blood pressure, pregnancy, and concomitant therapy. Efficacy was assessed by the investigators using the Response Evaluation Criteria in Solid Tumors revised (RECIST) version 1.1. This study was performed in compliance with Ministry of Health, Labour and Welfare (MHLW) Good Post-marketing Study Practice for drugs (MHLW Ministerial Ordinance No. 171, dated December 20, 2004). Patient data collected from this investigation were reported to MHLW according to the Pharmaceutical Affairs Law.

### Analysis plan

Safety analysis population included all eligible patients who received at least one dose of sunitinib. Efficacy analysis population included patients with at least one measurable lesion who underwent efficacy assessment. The primary safety endpoint of this analysis was the occurrence of adverse drug reactions during the 168-day observation period from the first administration of sunitinib. The incidence of notable adverse reactions with sunitinib was also examined. These notable adverse reactions included: (1) platelet count decreased, white blood cell count decreased, anemia, and other bone marrow suppression; (2) gastrointestinal disorders; (3) hypertension; (4) cutaneous symptom (hand–foot syndrome); (5) abnormal liver function; and (6) hypothyroidism.

The primary efficacy endpoint was ORR, defined as the percentage of patients in the efficacy analysis population who achieved complete response or partial response. Efficacy was assessed from initiation of sunitinib administration until the date of response determination. Subgroup ORR analyses by baseline factors were conducted and included: sex, age, weight, body surface area, body mass index, clinical symptoms of pNETs, presence/absence of metastasis (liver, lymph nodes, peritoneum, bone), general condition [Eastern Cooperative Oncology Group performance status (ECOG PS)], liver function disorder, renal impairment, history of previous treatment for pNETs, history of pharmacotherapy, and initial dose per day.

## Results

### Patients and treatments

Between August 2012 and September 2015, 62 patients were registered in 17 clinical centers. All 62 patients were included in the safety analysis and 51 patients were included in the efficacy analysis. Ten patients were excluded from the efficacy analysis due to indeterminate efficacy evaluation (*n* = 10) or not meeting criteria for efficacy (*n* = 1). Patient demographics and baseline characteristics are summarized in Table 1.

**Table 1:** Patient demographic and baseline characteristics

Characteristic, *n* (%)	Sunitinib (*N* = 62)
Sex	
Male	30 (48.4)
Female	32 (51.6)
Age, years	
< 65	42 (67.7)
≥ 65	20 (32.3)
Mean (SD)	57.2 (11.42)
Median (range)	57.0 (29–77)
Histologic classification	
Well differentiated	56 (90.3)
Other	6 (9.7)
Type of pNET	
Functional	
Insulinoma	3 (4.8)
Gastrinoma	7 (11.3)
Glucagonoma	2 (3.2)
Non-functional	51 (82.3)
Clinical symptoms of pNETs	18 (29.0)
Metastasis	
Liver	52 (83.9)
Lymph nodes	21 (33.9)
Peritoneum	8 (12.9)
Bone	9 (14.5)
ECOG PS	
0	38 (61.3)
1	21 (33.9)
≥ 2	3 (4.8)
Hepatic function disorder	11 (17.7)
Renal impairment	6 (9.7)
History of previous treatment for pNETs	
Surgical	39 (62.9)
Transarterial embolization	11 (17.7)
Transarterial chemo-embolization	18 (29.0)
Radiotherapy	8 (12.9)
Pharmacotherapy	54 (87.1)
Everolimus	42 (67.7)
Octreotide	35 (56.5)

*ECOG PS* Eastern Cooperative Oncology Group performance status, *pNETs* pancreatic neuroendocrine tumors, *SD* standard deviation

**Fig. 1:**
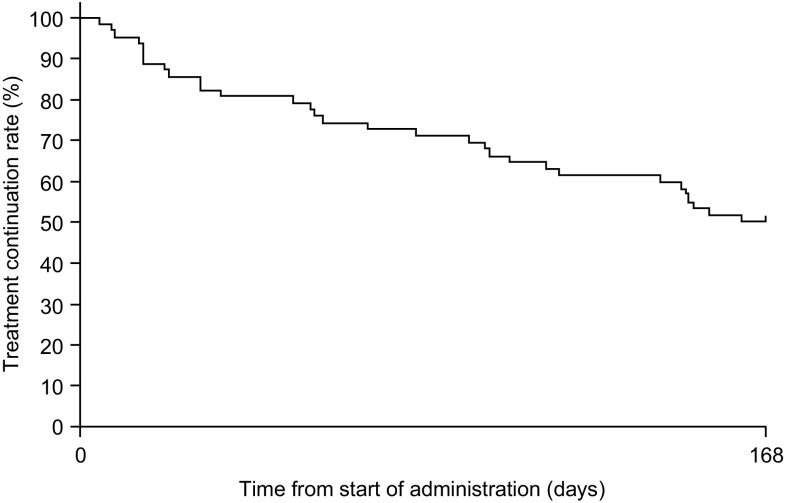
Duration of treatment

Sunitinib was administered for > 56 and ≤ 168 days in 48 (77.4%) patients and ≤ 56 days in 14 (22.6%) patients. Median (range) duration of treatment was 165 (4–168) days, and the mean ± SD total administered dose of sunitinib was 2711.49 ± 1722.159 mg. At 168 days from the start of treatment, 31 patients were still receiving sunitinib treatment and treatment continuation rate was 50.0% [95% confidence interval (CI) 37.1–61.6; Fig. 1]. Fourteen (22.6%) patients discontinued treatment ≤ 56 days, and 17 (35.4%) patients discontinued treatment between > 56 and ≤ 168 days. Discontinuation by duration of administration period is presented in Table 2.

**Table 2:** Discontinuation by duration of administration period

Administration period	Cumulative number of patients	Continuation of administration^a^		Reason for discontinuation^b^	
		Discontinuation	Proportion of accumulated patients (%)	Insufficient clinical effect^c^	Occurrence of AEs^c^
≤ 14 days	62	4	6.5	1	3
> 14 days to ≤ 42 days	58	8	13.8	1	7
> 42 days to ≤ 84 days	50	6	12.0	5	3
> 84 days to ≤ 126 days	44	6	13.6	4	2
> 126 days to ≤ 168 days^d^	38	7	18.4	5	3
Entire administration period	62	31	50.0	16	18

*AE* adverse event

^a^No patient had dose interruption

^b^No patient discontinued because of death or loss to follow up

^c^Patient could have had more than one reason for discontinuing

^d^The end of observation period of 168 days (24 weeks)

Of the 31 patients who discontinued treatment, 18 (58.1%) discontinued because of AEs occurrence and 16 (51.6%) due to insufficient clinical effect; patients could have had more than one reason for discontinuing. Of the patients who discontinued due to AEs, 10 discontinued within 42 days of treatment initiation.

The initial sunitinib dose was 37.5 mg/day in 43 (69.4%) patients, 25 mg/day in 16 (25.8%) patients, and 12.5 mg/day in three (4.8%) patients. Dose reduction occurred in 26 of 62 patients (41.9%), which included 23 of 43 patients administered a starting dose of 37.5 mg and three of 16 patients with starting dose of 25 mg. Per prescribing information, none of the 62 patients had cytokine P450 3A4 inhibitors co-administered with sunitinib [18].

### Safety

Overall, 300 all-grade AEs occurred in 59 (95.2%) patients and 57 grade ≥ 3 AEs occurred in 30 (48.4%) patients. The most commonly occurring all-grade AEs (Table 3) were platelet count decreased (33.9%), followed by diarrhea (29.0%), neutrophil count decreased (27.4%), hypertension (24.2%), and palmar-plantar erythrodysesthesia syndrome (24.2%). The most commonly (> 5% of patients) occurring grade ≥ 3 AEs (Table 3) were neutrophil count decreased (16.1%), platelet count decreased (14.5%), hypertension (6.5%), and white blood cell count decreased (6.5%).

**Table 3:** Adverse events occurring in ≥ 15% of patients

Preferred term for AE^a^, *n* (%)	Sunitinib (*N* = 62)		Median (range) time to onset, days
	All grades	Grade ≥ 3^b^	
Any AE	59 (95.2)	30 (48.4)	13.0 (1–119)
Platelet count decreased	21 (33.9)	9 (14.5)	20.0 (7–46)
Diarrhea	18 (29.0)	3 (4.8)	14.5 (3–149)
Neutrophil count decreased	17 (27.4)	10 (16.1)	27.0 (15–57)
Hypertension	15 (24.2)	4 (6.5)	15.0 (4–148)
PPE	15 (24.2)	2 (3.2)	36.0 (1–112)
White blood cell count decreased	12 (19.4)	4 (6.5)	23.0 (8–112)
Nausea	11 (17.7)	0	17.0 (4–126)
Abnormal liver function	11 (17.7)	0	20.0 (3–119)
Stomatitis	11 (17.7)	0	15.0 (6–43)
Decreased appetite	11 (17.7)	1 (1.6)	15.0 (8–126)

*AE* adverse event, *CTCAE* Common Terminology Criteria for Adverse Events, *MedDRA* Medical Dictionary for Regulatory Activities, *PPE* palmar-plantar erythrodysesthesia syndrome

^a^According to MedDRA (version 18.1) coding dictionary and CTCAE (version 4.0)

^b^There were no grade 5 AEs

AEs leading to permanent treatment discontinuation included: malaise (*n* = 3); decreased appetite, diarrhea, abnormal liver function, and platelet count decreased (*n* = 2, each); and influenza, hypothyroidism, heart failure, mitral insufficiency, aortic dissection, nausea, duodenal perforation, gastrointestinal perforation, vomiting, hepatic disorder, rash, fever, aspartate aminotransferase increased, alanine aminotransferase increased, and neutrophil count decreased (*n* = 1, each).

**Fig. 2:**
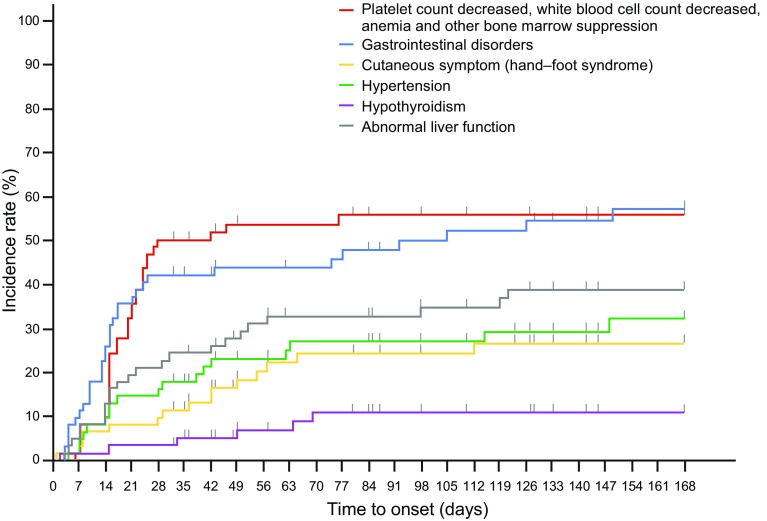
Time to onset of notable adverse reactions

Notable adverse reactions were first observed within 42 days (especially from day 14 to day 42) after the initiation of sunitinib administration (Fig. 2). There was no characteristic adverse reaction with a late-onset tendency. There were 22 serious AEs that occurred in 13 (21.0%) patients. The only serious AE occurring in two or more patients was diarrhea (two patients, 3.2%), the outcome of both of these AEs was disappeared/resolved.

### Efficacy

In the 51 patients eligible for the efficacy analysis, the ORR was 13.7% (95% CI 5.7–26.3). Clinical benefit rate, defined as complete response plus partial response plus stable disease, was 70.6% (95% CI 56.2–82.5; Table 4). The ORR analyzed according to ECOG PS before the start of sunitinib administration were 15.6% for patients with ECOG PS 0, 11.8% for patients with ECOG PS 1, and 0% for patients with ECOG PS 2 (Table 4). The response rate was 16.7% (*n* = 3) among 18 elderly (≥ 65 years old) patients and 12.1% (*n* = 4) among 33 non-elderly (< 65 years old) patients. Analysis by baseline factors revealed no major tendencies in efficacy.

**Table 4:** Best overall response, efficacy analysis set (*N* = 51)

ECOG PS	*n*	Best overall response evaluation					Response rate:		Disease progression control rate	
		CR	PR	SD	PD	Inconclusive	CR + PR (*n*)	95% CI	CR + PR + SD (*n*)	95% CI
–	51	0	7	29	14	1	13.7% (7)	5.7–26.3	70.6% (36)	56.2–82.5
By performance status^a^										
0	32	0	5	17	9	1	15.6% (5)	5.3–32.8	68.8% (22)	50.0–83.9
1	17	0	2	10	5	0	11.8% (2)	1.5–36.4	70.6% (12)	44.0–89.7
2	2	0	0	2	0	0	0% (0)	0.0–77.6	100% (2)	22.4–100.0

*CI* confidence interval, *CR* complete response, *ECOG PS* Eastern Cooperative Oncology Group performance status, *PD* progressive disease, *PR* partial response, *SD* stable disease

^a^There were no patients with ECOG PS 3–5

## Discussion

The results of this PMS study showed the safety profile of sunitinib in Japanese patients with pNETs treated in clinical practice was similar to the safety profile in patients treated in clinical trials, including the global phase III and IV trials and the phase II trial in Japanese patients [8, 11, 19]. Furthermore, there were no new safety concerns in sunitinib-treated Japanese patients with pNETs, and the commonly reported AEs were similar to those previously reported in clinical trials and surveillance studies of sunitinib in patients with metastatic renal cell carcinoma and gastrointestinal stromal tumors [20–24].

Notably, of the 31 patients who discontinued sunitinib treatment, 18 (58%) discontinued because of AEs; of these, 10 (56%) patients discontinued as early as within 42 days after the start of sunitinib administration. This suggests the Japanese clinicians decided on treatment discontinuation at the initial occurrence of AEs without considering temporary dose interruption or dose reduction to manage the AEs. As a result, the median duration of treatment was short (165 days) and ORR was low (13.7%). In fact, the median duration of treatment in this PMS study was shorter than in the global phase IV trial (356 days) or the real-world clinical setting of the Japanese clinical study (490 days) [19, 25]. The longer treatment duration in those trials likely led to the better outcomes: ORR was 24.5% in the global phase IV trial and 44% in the Japanese study [19, 25]. Keeping patients on treatment longer, by appropriately managing AEs at early stages, is essential for achieving better clinical outcome in sunitinib-treated patients with pNETs.

The most commonly reported AEs in this Japanese PMS study were similar to those reported in the phase III pivotal trial, the global IV study, and the phase II Japanese trial [8, 11, 19]. The most common AEs of bone marrow depression observed in this Japanese PMS study were lower than those reported in the global phase IV study and included neutrophil count decreased (neutropenia in the global phase IV) (27.4% vs. 53.8%) and white blood cell count decreased (leukopenia in the global phase IV) (19.4% vs. 43.4%), respectively [19]. One potential reason for the lower frequency of these AEs in this Japanese PMS study compared with the global phase IV trial is the shorter treatment duration (165 vs. 490 days, respectively) [19].

The AEs leading to treatment discontinuation included malaise, decreased appetite, diarrhea, abnormal liver function, and platelet count decreased, and most were of grade 1–2. Moreover, many of the AEs occurred between 14 and 42 days after the start of sunitinib administration. Therefore, anticipating the occurrence of AEs and their timing, and managing AEs by dose interruption/reduction could have resulted in longer duration of sunitinib treatment and better clinical outcome. In fact, as a result of AEs management by dose interruption/reduction in the Japanese phase II trial and Japanese real-world clinical setting, respectively, continued long-term administration of sunitinib (median, 298 days and 490 days) was achieved and ORR was high (50% and 44%) [11, 25].

In conclusion, there were no new safety concerns with sunitinib in this post-marketing study in Japanese patients with pNETs. Continuation of sunitinib administration for as long as possible leads to improved prognosis in patients with pNETs. Therefore, anticipation and management of AEs through appropriate dose interruption/reduction in actual clinical settings is essential for treatment success with sunitinib in patients with pNETs.
